# Sulindac Sulfide Reverses Aberrant Self-Renewal of Progenitor Cells Induced by the AML-Associated Fusion Proteins PML/RARα and PLZF/RARα

**DOI:** 10.1371/journal.pone.0022540

**Published:** 2011-07-19

**Authors:** Gunnar Steinert, Claudia Oancea, Jessica Roos, Heike Hagemeyer, Thorsten Maier, Martin Ruthardt, Elena Puccetti

**Affiliations:** 1 Department of Hematology, Goethe-University, Frankfurt, Germany; 2 Institute of Molecular Biology and Tumor Research, Philipps-Universität, Marburg, Germany; 3 Institute of Pharmaceutical Chemistry, Goethe-University, Frankfurt, Germany; University of Barcelona, Spain

## Abstract

Chromosomal translocations can lead to the formation of chimeric genes encoding fusion proteins such as PML/RARα, PLZF/RARα, and AML-1/ETO, which are able to induce and maintain acute myeloid leukemia (AML). One key mechanism in leukemogenesis is increased self renewal of leukemic stem cells via aberrant activation of the Wnt signaling pathway. Either X-RAR, PML/RARα and PLZF/RARα or AML-1/ETO activate Wnt signaling by upregulating γ-catenin and β-catenin. In a prospective study, a lower risk of leukemia was observed with aspirin use, which is consistent with numerous studies reporting an inverse association of aspirin with other cancers. Furthermore, a reduction in leukemia risk was associated with use of non-steroidal anti-inflammatory drug (NSAID), where the effects on AML risk was FAB subtype-specific. To better investigate whether NSAID treatment is effective, we used Sulindac Sulfide in X-RARα-positive progenitor cell models. Sulindac Sulfide (SSi) is a derivative of Sulindac, a NSAID known to inactivate Wnt signaling. We found that SSi downregulated both β-catenin and γ-catenin in X-RARα-expressing cells and reversed the leukemic phenotype by reducing stem cell capacity and increasing differentiation potential in X-RARα-positive HSCs. The data presented herein show that SSi inhibits the leukemic cell growth as well as hematopoietic progenitors cells (HPCs) expressing PML/RARα, and it indicates that Sulindac is a valid molecular therapeutic approach that should be further validated using *in vivo* leukemia models and in clinical settings.

## Introduction

More than 60% of acute myeloid leukemia (AML) cases involve specific chromosomal aberrations, mainly translocations. Ninety-seven percent of acute promyelocytic leukemia (APL) patients harbor the t(15;17) mutation, and less than 2% harbor the t(11;17) mutation, which leads to the formation of chimeric genes that encode the PML/RARα and PLZF/RARα (X-RARα) fusion proteins. X-RARα induce and maintain the leukemic phenotype by blocking terminal differentiation and increasing the self-renewal potential of leukemic stem cells (LSCs) [1], [2], [3]. One of the key mechanisms by which X-RARα increases LSC self-renewal is the activation of the Wnt signaling pathway [2], [4]. X-RARα activates Wnt signaling by upregulating γ-catenin and β-catenin at the transcriptional level [4]. Activation of Wnt signaling augments self-renewal of normal HSCs and LSCs [4], [5], [6].

Accumulation of β- and γ-catenin and subsequent transcriptional activation of T-cell-factor/lymphoid-enhancer-factor (TCF/LEF) family members facilitates the development of leukemia. Therefore, prevention of γ-catenin accumulation is an attractive target for chemotherapeutic-agents.

Activation of β-catenin plays a critical role in the development of colorectal cancer and has been implicated in prostate and breast cancer [7], [8], [9], [10], [11], [12], [13]. In the majority of these cases, aberrant activation of Wnt signaling depends on mutations in the APC or β-catenin genes that alter their interaction with axin and lead to release of β-catenin from its cytoplasmic destruction complex. In this complex, β-catenin is targeted for proteasomal degradation by phosphorylation via GSK3β [14], [15], [16]. Unphosphorylated β-catenin accumulates in the nucleus, where it associates with TCF/LEF family members and various transcription co-factors [17], [18], [19]. The resulting multi-protein complexes regulate expression of genes that are important for proliferation, differentiation, and apoptosis [13], [20].

Non-steroidal anti-inflammatory drugs (NSAIDs), such as ibuprofen, indomethacin, phenoprofen, and naproxen, inhibit the activity of β-catenin-dependent reporter genes in malignant cell lines and induce β-catenin degradation [21], [22], [23], [24], [25], [26], [27]. Moreover, the colonic polyps of patients treated with NSAIDs show reduced nuclear accumulation of β-catenin [21]. NSAIDs inhibit both β-catenin activity and stability [23], [24]. Nevertheless, the molecular mechanism of NSAID-mediated inhibition of β-catenin remains unclear.

Sulindac reduces the size and number of colorectal tumors in patients suffering from familial adenomatous polyposis, a pre-cancerous lesion that invariably develops into colon carcinoma if left untreated [28]. Sulindac metalabolizes into Sulindac sulfon (SSo) and Sulindac sulfide (SSi), both of which inhibit cell growth by inducing apoptosis in colon cancer cells. SSo has a more pronounced effect than SSi [29]. Notably, SSo exerts also a chemo-preventive effect on the development of colon cancer [30]. SSi is a dual COX-1/-2 inhibitor, whereas SSo does not show any inhibitory effect on COX enzymes. This result indicates that the effects of Sulindac are independent of its ability to inhibit COX-1 and COX-2.

Here, we investigated the effects of Sulindac derivatives on the X-RARα-induced leukemic phenotype to explore the potential of NSAIDs as a novel therapeutic approach for acute leukemia.

## Materials and Methods

### Cell lines and chemicals

NB4 and KG-1 cell lines were obtained from the German Resource Centre for Biological Material, Braunschweig, Germany and maintained in RPMI 1640 with 10% fetal calf serum (FCS)(Invitrogen, Karlsruhe, Germany). Additionally, the KG-1 cells were maintained in serum-free X-Vivo10 medium (Cambrex, Verviers, Belgium). Phoenix and 293 cells were cultured in DMEM with 10% FCS. SSi and SSo (Sigma, Steinheim, Germany) were dissolved in DMSO at a concentration of 73.4 mM and 146.9 mM, respectively, for stock solutions, and then appropriately diluted in the medium. Apoptosis was assessed by 7-amino-actinomycin (7AAD; Sigma) staining.

### Plasmids

The retroviral vectors PINCO-PML/RARα, PINCO-γ-catenin, and PINCO-PLZF/RARα were described previously[3]. PINCO-HA-β-catenin-S33A was created from pCMMBC-β-catenin-S33A (kindly provided by A. Starzinski-Powitz, Frankfurt University, Germany), using the Gateway system according to the manufacturer's instructions (Invitrogen). The TopFlash/FopFlash system and the *Renilla* luciferase pRL-TK construct (Promega, Mannheim, Germany) were described previously [31].

### Transactivation assays

The 293 cells were co-transfected with pCDNA3, pC3-PML/RARα, or pC2MMBC-β-catenin-S33A expression plasmids and pRT-LK, pGL3-OT, or pGL3-OF (the pGL3-OT promoter contains three TCF/LEF binding sites, whereas pGL3-OF contains mutated, inactive binding sites and is a negative control) using the calcium-phosphate method. Eight hours after transfection, each sample was subdivided and exposed either to 0.02% DMSO or 100 µM SSi. After an additional 24 h, the luciferase activity was determined using the “Dual-Luciferase Reporter Assay” according to the manufacturer's instructions (Promega). All assays were normalized to co-transfected *Renilla* activity.

### Western blotting

Western blotting was performed according to widely established protocols. The antibodies used were the following: anti-RARα, anti-γ-catenin (Santa Cruz Biotechnology, Santa Cruz, CA), anti-β-catenin (Cell Signaling, Frankfurt, Germany), anti-active β-catenin (Upstate, Charlottesville, VA, USA), anti-α-tubulin (NeoMarkers, Freemont, CA, USA), and anti-β-actin (Abcam, Cambridge, UK).

### Real-time PCR-TaqMan (qRT-PCR)

Total RNA and first strand DNA were obtained according to standard protocols. TaqMan-PCR was performed in triplicate using the ABI PRISM 7700 (Applied Biosystems, Foster City, CA, USA). The related “assays-on-demand” for LEF1, CyclinD1 and Axin2 transcripts were used according to the manufacturer's instructions (Assay-IDs: LEF1 - Mm0550265_m1; CyclinD1 - Mm0432359_m1; Axin2 - Mm00443610_m1)(Applied Biosystems). CT values were exported into an OpenOfficeCalc worksheet for calculation of fold changes using the comparative CT method. The target amount was normalized to glyceraldehyde-3-phosphate dehydrogenase (GAPDH) (Assay-ID: Mm99999915_g1)using the 2^-ΔΔCT^ method.

### Isolation of Sca1^+^/lin^−^ HPCs

Sca1^+^/lin^−^ HSCs were isolated from 8 12-week-old C57BL/6N female mice (Harlan, Borchen Germany). Bone marrow (BM) was harvested from femurs and tibiae by flushing the bones. The cells were “lineage depleted” using the “MACS Lineage Cell Depletion Kit” (Myltenyi, Bergisch Gladbach, Germany). Sca1^+^ cells were then purified by immunomagnetic beads using the “EasySep Mouse Sca1 Selection Cocktail” (StemCell Technologies) and pre-stimulated for 2 days in medium containing mIL-3 (20 ng/mL), mIL-6 (20 ng/mL), and mSCF (100 ng/mL)(Cell Concepts).

### Retroviral transduction

Phoenix packaging cells were transfected with retroviral vectors as described previously [3] 2 and 3 days post-transfection. Target cells were plated onto retronectin-coated (Takara-Shuzo, Shiga, Japan), non-tissue-culture 24-well plates and exposed to the retroviral supernatant for 3 hours at 37°C in the presence of 4 µg/ml polybrene (Sigma). The infection efficiency was at least 70%, as assessed by detection of GFP-positive cells.

### Replating efficiency, differentiation

At 5 days post-infection, Sca1^+^/lin^−^ cells were plated at 5×103 cells/mL in methylcellulose containing mIL-3 (20 ng/mL), mIL-6 (20 ng/mL), and mSCF (100 ng/mL) (StemCell Technologies) in the presence of either 0.02% DMSO or 100 µM SSi. The number of colonies was determined 10 days after plating. The cells were harvested by thorough washing, stained with specific antibodies for the detection of differentiation, and plated at 5×103 cells/plate in methylcellulose to determine replating efficiency via serial replating. Differentiation was assessed by expression of c-Kit, Sca1, Gr-1, and Mac-1 (BD/Pharmingen).

### Proliferation competition assay (PCA)

All animal studies were conducted in accordance with national animal protection laws (Regierungspräsidium Darmstadt - F 39/06). Sca1^+^/lin^−^ cells isolated from C57BL/6N mice were retrovirally transduced and cultivated in medium with 10% FCS, mIL-6, mSCF, and mIL-3 for 7 days upon exposure to either 40 µM SSi or 0.02% DMSO. At days 2, 4, and 7 after transduction, GFP expression was measured, and the GFP ratio between SSi- and vehicle-treated cells was calculated.

### Colony forming unit - spleen day 12 assay (CFU-S12)

In this study, 104 Sca1^+^/lin^−^ cells were retrovirally transduced, cultivated and treated as described above. The cells were then inoculated into lethally irradiated (11 Gy) recipients that were sacrificed 12 days later. Spleens were harvested and fixed with Tellysniczky's fixative. Colonies were counted as described previously [32].

### Competitive repopulation assay (CRA)

CD45.1^+/^Sca1^+^/lin^−^ cells that were retrovirally transduced, cultivated, and treated as described above were injected into lethally irradiated (11 Gy) CD45.2^+^ female recipients with 5×105 Ly5.2^+^ BM cells. The proportion of CD45.1^+^ donor cell-derived hematopoietic cells was determined using FACS of either BM or spleen MNCs.

### Statistical analysis

Statistical significance was determined by the unpaired Student's t-test and Log-rank (Mantel-Cox) test using the GraphPad Prism software (GraphPad, San Diego, CA). A p<0.05 value was considered significant.

## Results

### Sulindac derivatives downregulate β-catenin and γ-catenin, key mediators of Wnt signaling in PML/RARα-positive leukemic cells

**Figure 1:**
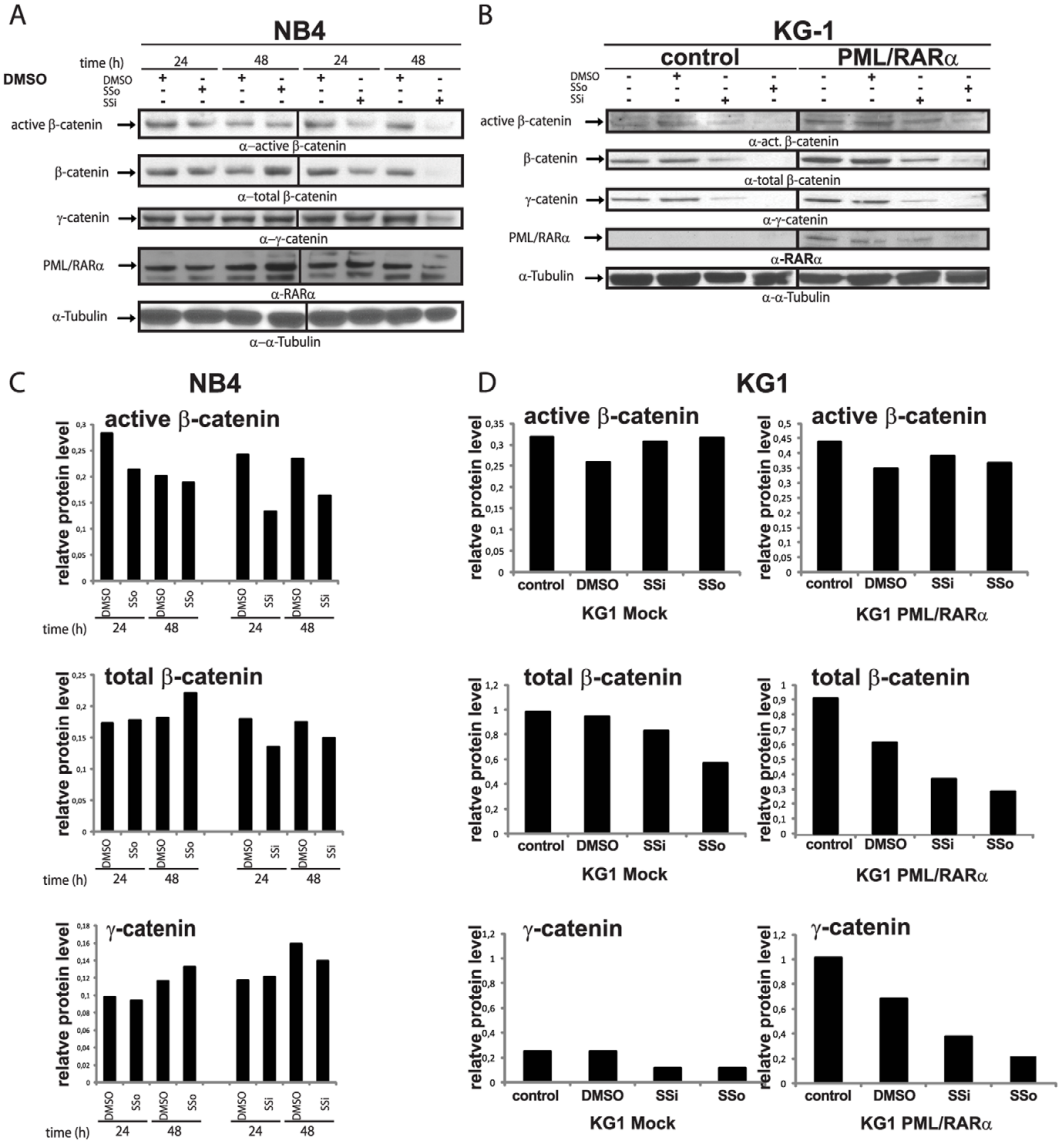
The effect of Sulindac derivatives on the expression of β-catenin and γ-catenin in PML/RARα-positive leukemic cells. Protein expression of active β-catenin, total β-catenin, γ-catenin and PML/RARα was assessed by western blot analysis with the indicated antibodies. (*A*) Patient-derived PML/RARα-positive NB4 cells treated with SSo (200 µM), SSi (200 µM) or 0.02% DMSO at 24 h and 48 h. (*B*) PML/RARα-expressing KG-1 cells treated at the cell type-specific IC_50_ (100 µM - SSi, 360 µM - SSo and 0.02% DMSO) at 72 h. Control - empty-vector-transfected cells; α-α-tubulin-loading control. (*C*–*D*) protein quantification of western blots presented in panel A and B, the bars represent the band intensity relative to the intensity of the tubulin bands reveled with a S-800 Densitometer and the Quantity One software (Bio-Rad).

X-RARα-mediated leukemogenesis is related to Wnt signaling activation by upregulation of β-catenin and γ-catenin [3], [4]. NSAIDs, such as Sulindac and its derivatives, are known to inhibit Wnt signaling in colon carcinoma cells by inducing β-catenin degradation [33]. Therefore, we wondered whether pharmacologically active Sulindac derivatives could also interfere with the leukemogenic potential of PML/RARα. We investigated the effects of Sulindac derivatives, Sulindac sulfon (SSo) and Sulindac Sulfide (SSi), on the expression levels of β-catenin and γ-catenin in patient-derived NB4 cells using western blot analysis. We found that SSo slightly reduced active β-catenin after 24 h, but neither total β-catenin nor γ-catenin. Conversely, SSi downregulated expression of total β-catenin and γ-catenin as well as active β-catenin starting at 24 h. This effect had the largest magnitude at 48 h. SSi (but not SSo) was also able to downregulate the expression of PML/RARα at 48 h (Figure 1A). Notably, SSo seemed to stabilize PML/RARα at 48 h.

To investigate whether the effects of the Sulindac derivatives were either 1) genetically determined by the presence of t(15;17) and thus mediated by PML/RARα, 2) a feature of promyelocytes, or 3) a general effect of transformed cells, we studied their effects on expression of β-catenin and γ-catenin in KG-1 cells stably transfected with PML/RARα compared with mock transfected KG-1 control cells. We exposed KG-1 cells to 360 µM SSo and 100 µM SSi (the specific IC50s for apoptosis induction, data not shown). In Figure 1B, we show that SSo and SSi downregulated active β-catenin as well as total β-catenin and γ-catenin in both control cells and PML/RARα-positive KG-1 cells.

In summary, these data show that SSi targets β-catenin and γ-catenin more efficiently than SSo, an effect apparently not mediated by PML/RARα.

### Effects of SSi (but not SSo) on APL cells occur at clinically relevant dosages

**Figure 2:**
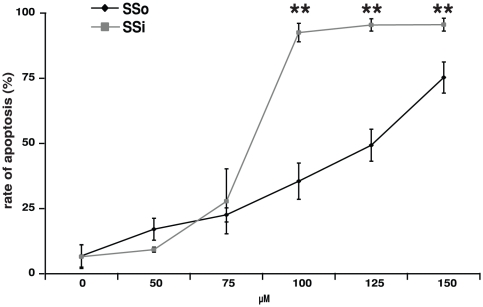
Induction of apoptosis in patient-derived NB4 cells by Sulindac derivatives. Rate of apoptosis in patient-derived PML/RARα-positive NB4 cells was assessed by 7AAD staining upon exposure to clinically achievable concentrations (50–150 µM) of SSi and SSo. Apoptosis was measured after 72 h. Data are expressed as the mean of three independent experiments with standard deviation (SD). Statistical analysis was performed using Student's *t*-test (* - p<0.05; ** - p<0.01).

The maximum plasma concentrations after either SSo or SSi oral administration to patients were 100 µM and 50–100 µM, respectively [21], [34], [35]. To examine clinical doses of SSo and SSi, we compared the capacity of these compounds to induce apoptosis in NB4 cells at concentrations between 0 and 150 µM. As shown in Figure 2, SSi induced physiologically relevant rates of apoptosis within clinically achievable concentrations, between 75 and 100 µM, whereas 100 µM SSo induced only very limited apoptosis. Based on these data, all subsequent studies were performed using SSi.

### SSi interferes with the aberrant replating efficiency of X-RARα-expressing HSCs and that induced by active β-catenin and γ-catenin

**Figure 3:**
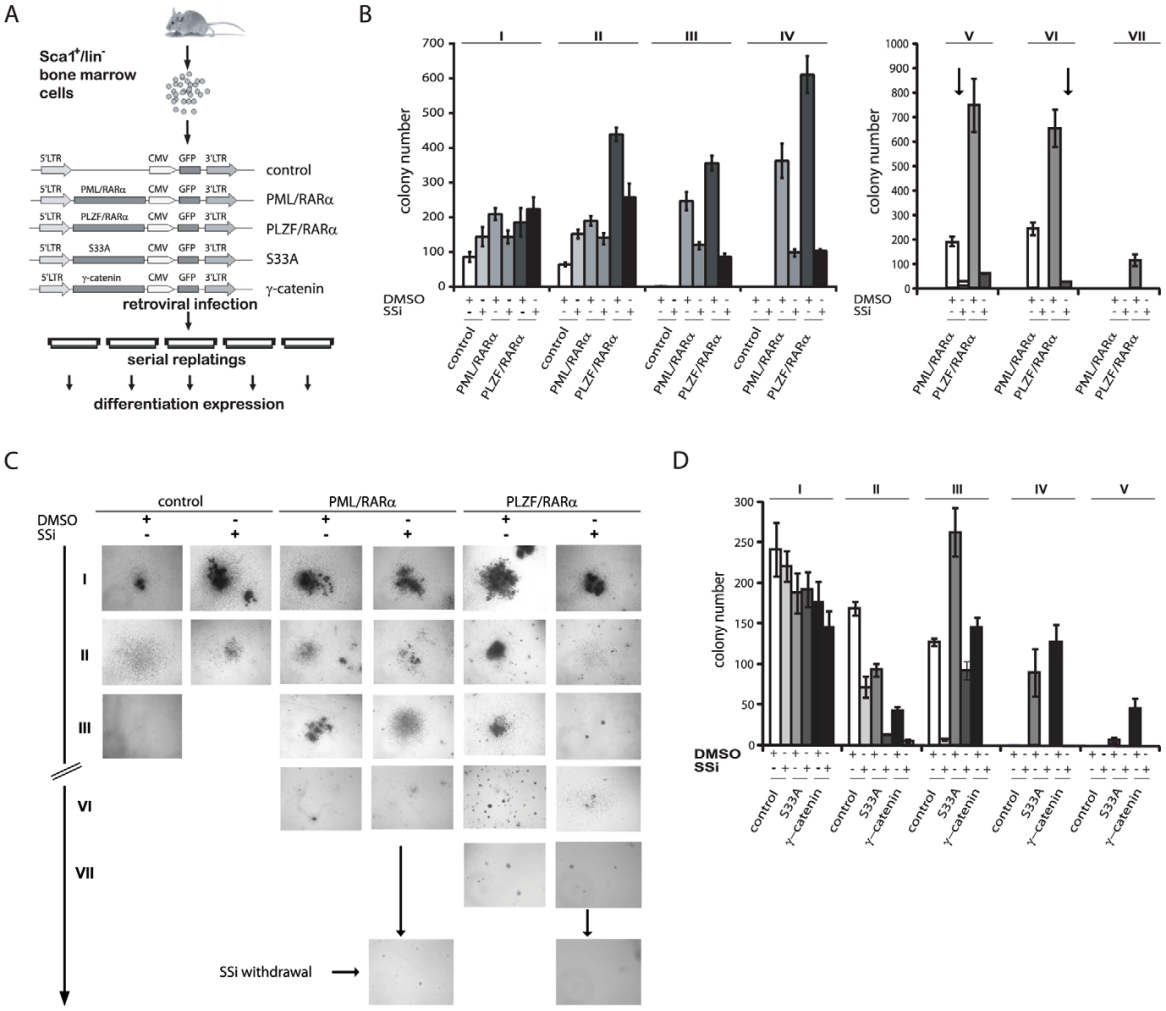
Effect of SSi on the biology of X-RARα-, γ-catenin- and constitutively active β-catenin-expressing murine HSCs. (*A*) Experimental strategy. Sca1^+^/lin^-^ BM cells were infected with the indicated retroviruses and plated in semi-solid medium with the indicated growth factors to determine the serial plating potential in the presence of either 100 µM SSi or 0.02% DMSO. (*B*) Long-term serial replating (I–VII) of HSC and X-RARα-positive HSC. SSi withdrawal after plating round V for PML/RARα-positive colonies and after plating round VI for PLZF/RARα-positive colonies. (*C*) Colony morphology of plating rounds I, III, VI, and VII as well as SSi withdrawal. (*D*) Long-term serial replating (I–V) of γ-catenin- and β-catenin-S33A-positive HSC. Data are expressed as the mean of three independent experiments with SD. Control - empty vector; ↓-SSi withdrawal; I–VII: serial plating rounds.

Activated Wnt signaling is considered to be primarily responsible for the aberrant biology of X-RARα-expressing HSCs. Therefore, we studied the effects of SSi on the replating efficiency of X-RARα-positive Sca1^+^/lin^−^ HPCs (Figure 3A). Here, we report that SSi reduced not only the CFU number, but also the number of serial replating rounds possible with cells expressing X-RARα. These cells could only be replated 5 times, in contrast with control cells. Withdrawal of SSi did not recover the replating potency of HPCs expressing X-RARα, thus indicating that the effect of SSi is irreversible (Figure 3B). The morphology of the colonies was classified as type A, B, or C, according to Lavau and co-workers [36]. In the first two rounds of plating, X-RARα-positive cells displayed mainly type B colonies, with a dense center surrounded by a halo of migrating cells (Figure 3C). Upon further serial replatings, small, compact, typical types A colonies, known to represent immature cells, were dominant [3]. Upon treatment with SSi, the X-RARα-positive colonies exhibited a clear type C morphology of diffuse colonies with mobile differentiating cells, revealing a differentiation-inducing effect of SSi (Figure 3C).

Expression of γ-catenin increases the serial replating potential of murine HSCs [3], and activation of β-catenin by Wnt ligands induces increased self-renewal [5], [37]. To determine whether SSi inhibits these effects, we exposed Sca1^+^/lin^−^ HPCs, transduced with either constitutively active β-catenin (S33A) or γ-catenin, to SSi. The S33A mutant harbors four mutated phosphorylation sites (S33, S37, S41, S45), preventing its proteasomal degradation and leading to constitutively active β-catenin. SSi decreased the number of CFUs in single plating rounds and the overall replating efficiency for HPCs expressing either active β-catenin or γ-catenin (Figure 3D).

In summary, these data provide evidence that SSi abrogates the leukemogenic potential of X-RARα. Furthermore, SSi reversed the effects of both β-catenin and γ-catenin on HPCs.

### SSi reduces Wnt signaling in PML/RARα-expressing cells

**Figure 4:**
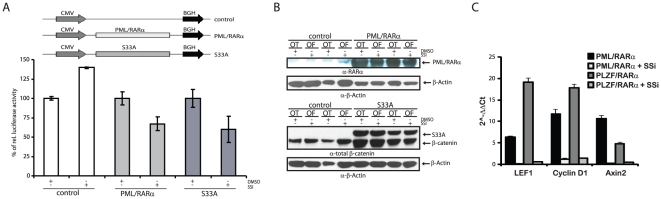
Effect of SSi on PML/RARα-mediated Wnt signaling pathway activation. (*A*) Transactivation assay for Wnt-signaling-related TCF/LEF-dependent transcription. Indicated expression vectors were co-transfected with either Topflash (OT) or Fopflash (OF) (the pGL3-OT promoter contains three TCF/LEF binding sites, whereas pGL3-OF contains mutated inactive binding sites and is a negative control) reporter constructs into 293 cells and exposed them to either 100 µM SSi or 0.02% DMSO. After 48 h, luciferase activity was measured and normalized to co-transfected *Renilla* activity. (*B*) Western blots for the expression of PML/RARα and S33A using the indicated antibodies, α-β-actin-loading control. (*C*) Effects of SSi on Wnt target genes in X-RARα-positive Sca1^+^/lin^-^ HPCs. Results are represented as 2^-ΔΔCT^ values. Each experiment was performed three times in triplicate, with similar results obtained each time. One representative experiment with SD is shown.

That PML/RARα induces activation of Wnt signaling similar to Wnt ligands and increases HSC self-renewal prompted us to ask whether SSi interferes with Wnt signaling activation by PML/RARα, assessed by TCF/LEF-dependent transactivation. Therefore, we transduced 293 cells with PML/RARα and S33A as a positive control (Figure 4A and 4B) and assessed Wnt signaling activation by luciferase activity using the Topflash/Fopflash system. PML/RARα and S33A induced promoter activation, which decreased upon treatment with SSi (Figure 4A). Furthermore, we used qRT-PCR to investigate the effect of SSi on Wnt signaling in X-RARα-positive Sca1^+^/lin^−^ HPCs via expression of the Wnt target genes, LEF1, CyclinD1 and Axin2, in the presence/absence of 40 µM SSi. As shown in Figure 4C, expression of LEF1, cyclin D1 and Axin2 was inhibited by SSi treatment in X-RARα-expressing Sca1^+^/lin^−^ HPCs. Taken together, these data show that SSi reduce PML/RARα-mediated aberrant activation of Wnt-signaling and downregulate Wnt-specific target genes.

### SSi reverses the X-RARα-induced differentiation block in murine HSCs

**Figure 5:**
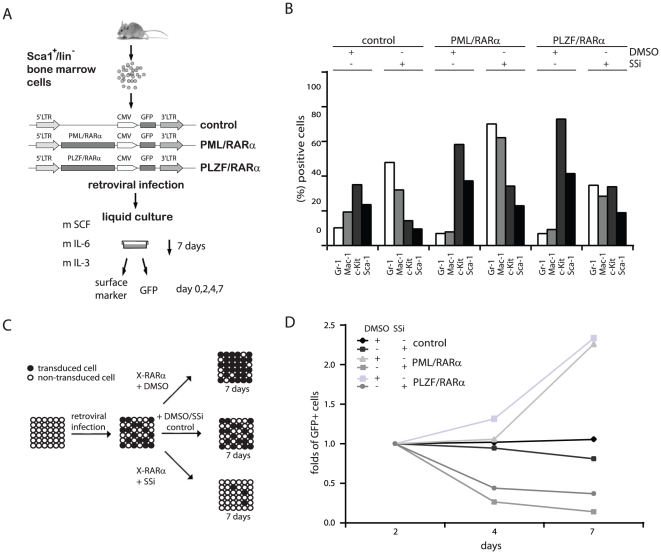
Effect of SSi on the X-RARα-induced phenotype in murine HSCs. (*A*) Experimental strategy. Sca1+/lin^-^ BM cells were infected with the indicated retroviruses and maintained for one week in liquid culture with the indicated growth factors in the presence/absence of 40 µM SSi. (*B*) Differentiation was assessed by the expression of Gr-1, Mac-1, c-Kit and Sca1. (*C*) Schematic representation of the proliferation competition assay (PCA). (*D*) GFP expression in X-RARα-positive HPCs assessed by FACS analysis in the presence/absence of SSi at day 2, 4, and 7. One representative result from three independent experiments is shown.

One feature of the leukemic phenotype is inhibited differentiation of early hematopoietic precursors. X-RARα inhibits terminal differentiation in murine HSCs [3]. To study whether the effects of SSi on replating efficiency leukemic HPCs were caused by reversal of the differentiation block, we investigated the effects of clinical doses of SSi on differentiation of X-RARα-expressing Sca1^+^/lin^−^ HPCs (Figure 5A). Differentiation was assessed by expression of specific surface markers such as Gr-1, Mac-1, Sca1, and c-Kit. We performed these experiments in liquid culture to demonstrate the effects of SSi on mock-transduced control HPCs, which should fully differentiate spontaneously in semi-solid medium. We found that SSi induced differentiation despite the presence of X-RARα, as demonstrated by an increase in Gr-1^+^ and Mac-1^+^ and decrease in Sca1^+^ and c-Kit^+^ cells (Figure 5B). To assess whether SSi-induced differentiation interfered with HSC proliferation, we performed PCA experiments that compared X-RARα-expressing HPCs to an empty vector control. The principle of the assay is depicted in Figure 5C; HPCs were transduced with the PINCO constructs, in which the LTR drives transgene expression. GFP expression is driven by a CMV promoter. The effect of SSi was demonstrated by detecting GFP-positive cells using FACS at 1 week. We found that SSi exposure led to reduced proliferation of X-RARα-positive HPCs, with no such effect on control cells (Figure 5D).

In summary, we provide evidence that SSi reverses the leukemic differentiation block and decreases X-RARα-dependent proliferation.

### SSi inhibits the short-term stem cell capacity of PML/RARα- and PLZF/RARα-positive HPCs

**Figure 6:**
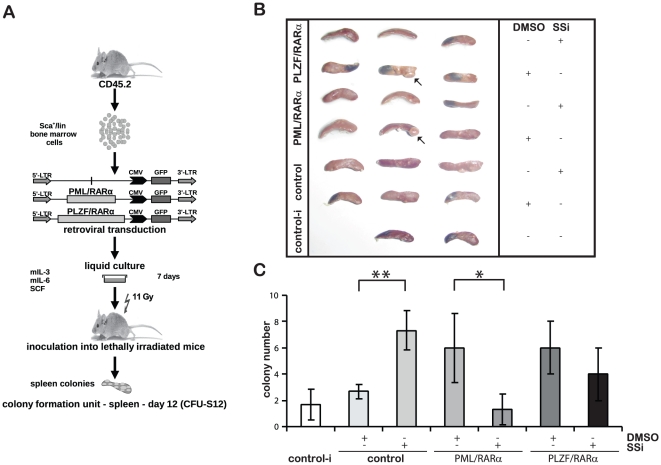
Effect of SSi on X-RARα-mediated aberrant stem cell capacity in murine HSC. (*A*) Experimental strategy for studying the ST-HSC capacity of X-RARα-expressing HSCs (CFU-S12). Sca1^+^/lin^-^ BM cells were infected with the indicated retroviruses and maintained for one week in liquid culture with the indicated growth factors in the presence/absence of 40 µM SSi. Cells were inoculated into lethally irradiated (11Gy) recipients that were then sacrificed at day 12 after transplantation. (*B*) Photographs of all the fixed spleen are shown, the arrows indicate an example of a CFU in the fixed spleens. (*C*) Bulks represent the number of CFU-S12 expressed as the mean from three spleens with SD. Statistical analysis was performed using Student's *t*-test (* - p<0.05; ** - p<0.01).

Our data suggest that SSi interferes with the stem cell maintenance capacity of X-RARα-positive HPCs. To confirm this mechanism, we performed a CFU-S12 assay on X-RARα-positive Sca1^+^/lin^−^ HPCs cultured for seven days in the presence/absence of 40 µM SSi, to detect “short-term repopulating stem cells” (ST-HSC) (Figure 6A) [38]. We found that the CFU-S12 number for X-RARα-positive HPCs was reduced upon exposure to SSi (Figure 6B-C). The arrows indicate an example of a CFU in the fixed spleens (Figure 6B), the number of CFUs is represented in a bar graph (Figure 6C). The trend was not statistically significant in PLZF/RARα-transduced cells, in contrast with PML/RARα-positive HPCs; however, a trend toward a reduced number of CFU-S12 was identified. Of note, we also observed a “paradoxical” effect of SSi on empty vector controls, as demonstrated by an increased number of CFU-S12s compared with vehicle-treated cells (Figure 6B–C).

Taken together, these data show that SSi reduces the ST-HSC capacity of X-RARα-positive HPCs.

### SSi inhibits the long-term stem cell capacity of PML/RARα-positive HPCs

**Figure 7:**
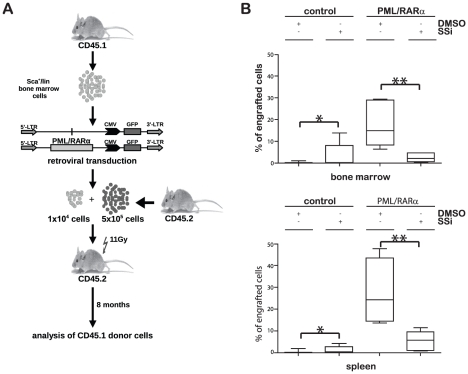
Effect of SSi on PML/RARα-mediated aberrant long-term stem cell capacity in murine HSC. (*A*) Experimental strategy for studying the LT-HSC capacity of PML/RARα-expressing HSCs. Sca1^+^/lin^-^ BM cells from CD45.1^+^ mice were infected with the indicated retroviruses and maintained for one week in liquid culture with the indicated growth factors in the presence/absence of 40 µM SSi. The cells were co-transplanted with CD45.2^+^ BM cells into lethally (11 Gy) irradiated CD45.2^+^ recipient mice. (*B*) Donor chimerism 8 months after transplantation was used to determine LT-HSC capacity in the BM and spleen. Statistical significance was determined using the Log-rank (Mantel-Cox) test (* <0.05; ** <0.01).

To determine whether SSi interferes with the capacity of X-RARα to maintain long-term stem cell (LT-HSC) capacity, we performed a CRA assay [38] on PML/RARα-positive Sca1^+^/lin^−^ HPCs cultured for seven days in the presence/absence of 40 µM SSi (Figure 7A). The population density of LT-HSC was assessed via CD45.1/CD45.2 chimerism, which demonstrates the capacity of the PML/RARα-positive CD45.1^+^ donor cells to compete the reconstitution of hematopoiesis by CD45.2^+^ BM cells in lethally irradiated CD45.2^+^ recipients (Figure 7A). Upon exposure to SSi, we found that after 8 months, the proportion of PML/RARα-positive HPCs was significantly reduced in the BM and spleens of recipient mice compared with untreated controls (Figure 7B).

Taken together, these data show that SSi reduces the LT-HSC capacity of PML/RARα-positive HPCs via inhibition of Wnt signaling.

## Discussion

The aim of this study was to explore the possibility of treating leukemia cells with NSAIDs, which are known to inhibit Wnt signaling in several solid tumor models [21], [23], [24], [39]. Aberrant Wnt signaling is a key event during pathogenesis of APL and other leukemia [4], [6], [40], [41].

Herein, we show for the first time that the NSAID, Sulindac, interferes with the X-RARα-induced leukemic phenotype. These effects include degradation of the key Wnt signaling mediators β- and γ-catenin and overcoming the inhibition of differentiation and aberrant stem cell capacity.

In a prospective study, a lower risk of leukemia was associated with the use of aspirin [42] consistent with numerous studies reporting an inverse association of aspirin with other cancers. Furthermore, the use of other NSAIDs demonstrated a reduction in AML incidence, depending on the FAB classification [43]. Cell lines such as KG1 (a cell line derived from an AML patient FAB M2) show a sensitivity to Sulindac, independent of PML/RARα. These data are consistent with the data showing that the use of NSAIDs prevents this type of AML [43].

Furthermore, we show in our in vivo experiments that SSi dramatically reduces the short- and long-term repopulating efficiency of PML/RARα expressing HPCs, accompanied by inhibition of wnt signaling target genes such as LEF, CyclinD1, and Axin2. The data herein strongly suggest a novel mechanism for Sulindac in leukemia compared with its mechanism in either colon carcinoma or other solid tumors. We found that SSi had a more pronounced effect than SSo, in contrast to the more pronounced effect of SSo observed in colon carcinoma [29]. All the effects of SSi on either differentiation or stem cell capacity were demonstrated using clinical doses. Furthermore, we found that SSi had a stronger effect on the expression of β- and γ-catenin compared with SSo, which also differs from observations in colon carcinoma cells. Although both compounds diminished β- and γ-catenin expression, it is possible that the differences between SSi and SSo were due to different efficiencies in downregulating β- and γ-catenin that were not detectable in our models. In colon carcinoma cells, SSo is a known specific inhibitor of c-GMP phosphodiesterase, which leads to increased c-GMP levels, followed by activation of c-GMP-dependent kinases [44]. One consequence of this could be alternative phosphorylation of β- and/or γ-catenin, directing these proteins to proteasomal degradation [29], [33]. Conversely, SSi-induced degradation of β- and γ-catenin has been described as independent of its COX-1/-2 inhibitory activity. The prominent COX-independent activities associated with SSi are Raf inhibition and caspase activation [45], [46]. Raf inhibition can be excluded as the mechanism for inhibition of the leukemic phenotype because Ras signaling is apparently not involved in X-RARα-related leukemogenesis. Notably, β-catenin is known to stabilize COX-2 mRNA [47]. Thus, the downregulation of β-catenin accompanied by parallel inhibition of COX-2 might explain the stronger activity of SSi compared with SSo in our models. Hence, our data indirectly demonstrate that the effects of Sulindac derivatives on leukemic cells via COX inhibitory activity cannot be completely excluded, as SSo (not SSi) does not inhibit COX enzymes. Furthermore, SSi showed an effect at a concentration in which it inhibits not only COX but also the 5 lipoxygenase (5-LO), another key enzyme in the arachidonic acid cascade [48].

SSi might exhibit a stronger effect on APL cells due to the degradation of PML/RARα as well as β- and γ-catenin in NB4 cells. Notably, SSi was also able to decrease the biological activity of mutant β-catenin in murine HSCs, as evidenced by decreased TCF/LEF-dependent transcription. This common effect can be explained, by the downregulation of β-catenin by SSi via phosphorylation-independent mechanisms [49]. These effects may be mediated by SIAH upregulation, which is known to be involved in degradation of PML/RARα [50] and leads to loss of the oncogenic agent responsible for initiation and maintenance of the leukemic phenotype. Herein, we show that the combination of PML/RARα and SSI activates the SIAH promoter (Figure S1 and Data S1).

Recent data demonstrate that PML/RARα-induced self-renewal of HSCs is mediated by the induction of p21^cip1/waf1^, and the synergism of both is essential for the maintenance of leukemic stem cells [2].

The combined effects of SSi on Wnt signaling and PML/RARα suggest a great potential for SSi as a novel therapeutic approach in leukemic stem cell therapy. Supporting its therapeutic use, clinical concentrations of SSi reversed the X-RARα-related differentiation block and aberrant stem cell capacity. Notably, exposure to SSi led to an increase in stem cell capacity for normal HSCs. This effect in normal cells might be due to SSi-induced ERK1/2 activation, which has been shown to overcome SSi-related cytotoxicity [51].

That SSi does not inhibit Wnt signaling selectively in X-RARα-positive cells is not of high clinical importance because the activation of Wnt signaling is only indispensable for LSCs, not for normal HSCs [3], [52], [53] (Wang et al. 2010).

The clinical significance of our findings establishes that Sulindac derivatives are not only chemo-preventatives in colon carcinoma but also potentially novel compounds for use in maintenance therapy to control residual disease. This finding is consistent with recent data on the effects of NSAIDs in a MLL/AF9-induced leukemia model [41]. In fact, the principal aim of maintenance therapy is to control leukemic stem cells with compounds that have few undesired side effects. Sulindac derivatives have long half-lives, and renal and gastric toxicity can be easily controlled.

In summary, we provide a proof of principle that NSAIDs can be used for “stem cell therapy” approaches in leukemia and function by targeting Wnt signaling, which should be further validated in *in vivo* leukemia models and clinical settings.

## Supporting Information

Figure S1 **Transactivation of the SIAH1 promoter.** The indicated transgenes were co-transfected with the SIAH1 promoter fragment -297-0 into 293 cells and exposed to either 100 µM SSi or 0.02% DMSO. The data are the mean from two triplicate experiments with SD (Data S1).
(TIF)

Data S1 **Supporting information**
(DOC)
