# Supplementary figures and images for: Sulindac Sulfide Reverses Aberrant Self-Renewal of Progenitor Cells Induced by the AML-Associated Fusion Proteins PML/RARα and PLZF/RARα
Source: PLoS One. 2011 Jul 19;6(7):e22540. doi: 10.1371/journal.pone.0022540 (PMC3139642; doi:10.1371/journal.pone.0022540)

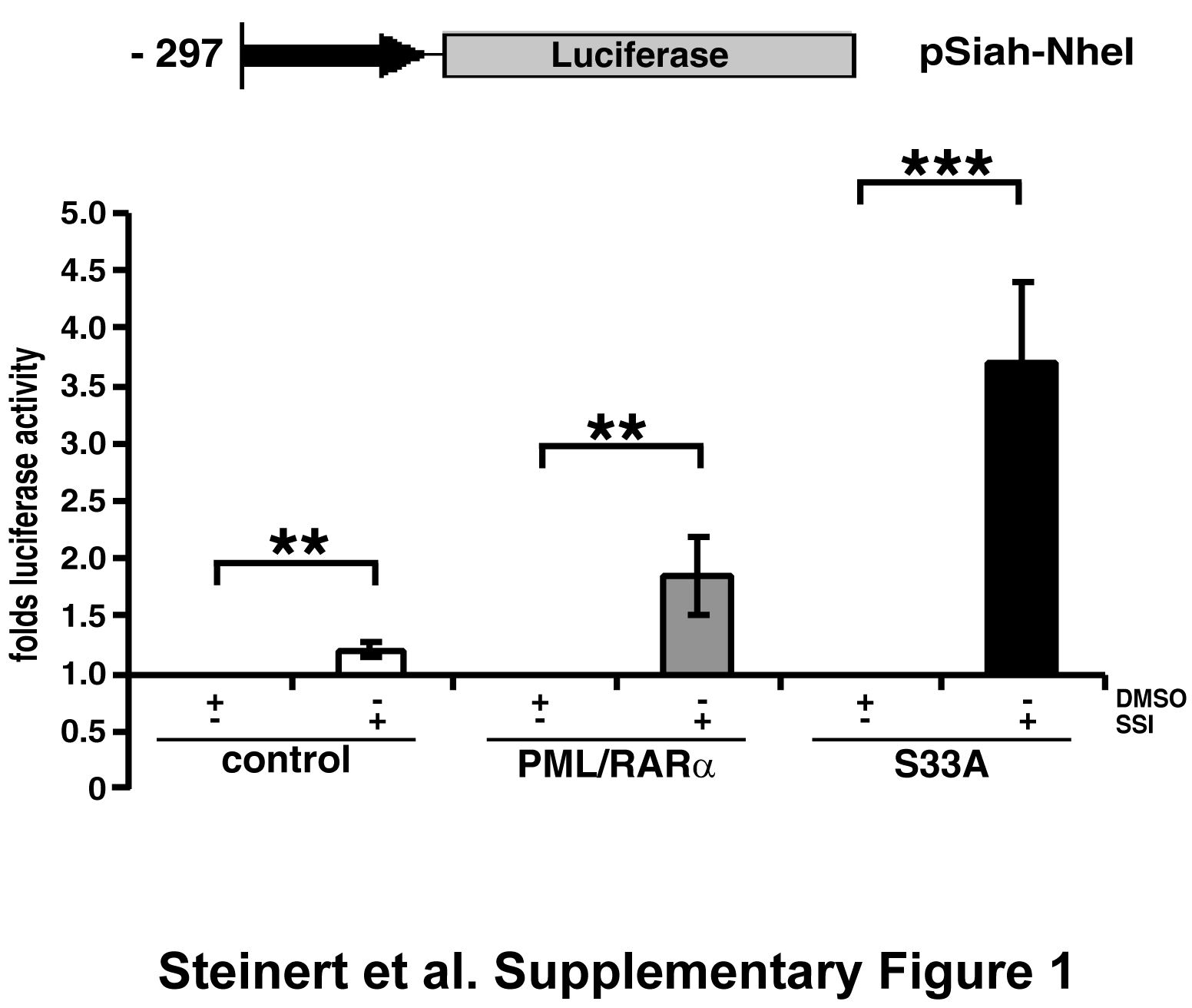

Supplement: Figure S1 — Transactivation of the SIAH1 promoter. The indicated transgenes were co-transfected with the SIAH1 promoter fragment -297-0 into 293 cells and exposed to either 100 µM SSi or 0.02% DMSO. The data are the mean from two triplicate experiments with SD (Data S1). (TIF) [file pone.0022540.s001.tif]
