# Supplementary material for: Sulindac Sulfide Reverses Aberrant Self-Renewal of Progenitor Cells Induced by the AML-Associated Fusion Proteins PML/RARα and PLZF/RARα
Source: PLoS One. 2011 Jul 19;6(7):e22540. doi: 10.1371/journal.pone.0022540 (PMC3139642; doi:10.1371/journal.pone.0022540)
Supplement: Data S1 — Supporting information (DOC) [file pone.0022540.s002.doc]

**Supplementary data**

**Supplementary Materials and Methods**

**Plasmids**

The SIAH1 promoter construct (pSIAH-NheI-279-luciferase) was kindly provided by T. Sakai (Kyoto Prefectural University of Medicine, Japan).

**Transactivation assays**

293 cells were co-transfected with pSIAH-NheI-279-luciferase, using the calcium-phosphate method. Eight hours after transfection, each sample was subdivided and exposed either to 0.02% DMSO or to 100 µM SSi. After an additional 24h, the luciferase activity was determined using the “Dual-Luciferase Reporter Assay” according to the manufacturer’s instructions (Promega). All assays were normalized to co-transfected *Renilla* activity.

**Supplementary Results**

Recently, an alternative phosphorylation-independent degradation pathway for β-catenin via SIAH1 was described [1]. SIAH proteins are also known to degrade PML/RARα [2]. To investigate whether alternative degradation pathways may mediate the activity of SSi, we studied the effects of SSi on SIAH1 promoter activity in the presence of either PML/RARα or S33A. We found that SSi led to a slight activation of the SIAH1 promoter in empty-vector controls, whereas greater activation was achieved in the presence of PML/RARα and mutant β-catenin (Supplementary Figure 1).

Taken together, these data show that SSi reduces PML/RARα-mediated aberrant activation of Wnt-signaling and leads to activation of the SIAH1 promoter, which may contribute to the effects observed.

**Bibliography**

1. Matsuzawa SI, Reed JC (2001) Siah-1, SIP, and Ebi collaborate in a novel pathway for beta-catenin degradation linked to p53 responses. Mol Cell 7: 915-926.

2. Fanelli M, Fantozzi A, De Luca P, Caprodossi S, Matsuzawa S, et al. (2004) The coiled-coil domain is the structural determinant for mammalian homologues of Drosophila Sina-mediated degradation of promyelocytic leukemia protein and other tripartite motif proteins by the proteasome. J Biol Chem 279: 5374-5379.

**Figure Legend**

**Supplementary Figure 1**

Transactivation of the SIAH1 promoter. Indicated transgenes were co-transfected with the SIAH1 promoter fragment -297-0 into 293 cells and exposed to 100 µM SSi or 0.02% DMSO. Data represent means of two experiments in triplicates with SD.
